# Indicators of profound hematologic response in AL amyloidosis: complete response remains the goal of therapy

**DOI:** 10.1038/s41408-020-00355-6

**Published:** 2020-09-01

**Authors:** Paolo Milani, Marco Basset, Mario Nuvolone, Francesca Benigna, Lara Rodigari, Francesca Lavatelli, Andrea Foli, Giampaolo Merlini, Giovanni Palladini

**Affiliations:** Amyloidosis Research and Treatment Center, Foundation IRCCS Policlinico San Matteo, and Department of Molecular Medicine, University of Pavia, Pavia, Italy

**Keywords:** Medical research, Prognosis

## Abstract

In AL amyloidosis complete response (aCR) is defined as negative serum and urine immunofixation with normalized free light chain ratio (FLCR). However, achievement of low levels of involved FLC (iFLC) or difference between iFLC and uninvolved FLC (dFLC) are also relevant endpoints for treatment. We divided 434 consecutive patients with AL amyloidosis into five groups according to response 6 months after treatment initiation: aCR, iFLC <20 mg/L, normalized-iFLC, dFLC <10 mg/L, and normalized FLC ratio. Overall survival (OS) was similar (median not reached) in patients in aCR and in those who reached iFLC <20 mg/L, while it was inferior in all other groups (medians ranging from 79 to 91 months). Time to next therapy or death (TNTD) was longer in subjects attaining aCR (median 69 months) than in subjects reaching any FLC endpoint (medians ranging from 18 to 39 months). The ability of discriminating patients who survived more than 2 years among all responders was greater for current definition of aCR compared to combination of negative serum and urine immunofixation with any low-FLC endpoint. Complete response predicts best outcomes in AL amyloidosis and should be the goal of therapy if tolerability allows.

## Introduction

Light chain (AL) amyloidosis is caused by a small plasma cell clone producing light chains that form amyloid deposit while causing organ dysfunction and damage^1^. Chemotherapy targeting the plasma cell clone aims at prolonging survival by obtaining deep reductions of the amyloid light chain. A study of the International Society of Amyloidosis involving 1190 patients, identified and validated hematologic response criteria that sharply discriminate groups with different overall survival (OS)^2^. Amyloid complete response (aCR) was defined as negative serum and urine immunofixation and normalized free light chain (FLC) ratio^2^. This predicted the longest OS in the testing and in the validation cohorts^2^. Other response categories were very good partial response (VGPR), defined as a post-treatment difference between involved (iFLC) and uninvolved FLC (dFLC) <40 mg/L, and partial response (PR) defined as a decrease of dFLC >50% compared to baseline^2^. The criteria eventually adopted in this international study were chosen based on their discriminating ability and validated in two independent populations based on OS, which was significantly longer for VGPR over PR and for aCR over VGPR^2^.

Several groups reported that in patients with low-FLC burden (a baseline dFLC <50 mg/L), achieving a difference between involved (iFLC) and uninvolved FLC (dFLC) <10 mg/L was associated with prolonged OS^3–5^. More recently, Manwani et al. showed that a dFLC <10 mg/L was a meaningful endpoint in all patients and, importantly, was associated with prolonged survival amongst subjects in aCR^6^. Furthermore, Mayo Clinic investigators reported that patients who obtain an iFLC concentration <20 mg/L or a normalization of iFLC had longer progression-free survival and were more likely to achieve organ response^7,8^. Lastly, Godara et al. recently noticed that patients achieving an iFLC <10 mg/L for any period of time post-therapy had over 90% survival at ten years^9^. Altogether, these recent reports started a lively debate on the opportunity to modify the currently validated definition of aCR. However, available evidence (1) does not prove the ability of criteria based on low-FLC levels to identify patients who do not satisfy the validated definition of aCR but whose OS is comparable to that of patients who qualify for aCR, (2) does not prove the ability of criteria based on low-FLC levels to identify patients with superior survival amongst those who attain aCR, and (3) lacks external validation. This information would be the minimum essential requirement to update the currently validated hematologic response criteria for AL amyloidosis. In the present study, we address these relevant questions in a series of patients mostly treated with nontransplant chemotherapy. A parallel study from the Boston group was performed in an independent cohort of patients undergoing autologous stem cell transplant.

## Subjects and methods

The prospectively maintained database including 1378 patients with AL amyloidosis newly diagnosed at the Amyloidosis Research and Treatment Center of Pavia (Italy), between 2004 and 2018 was systematically searched. Patients were included in our study if they reached aCR 6 months after treatment initiation, defined as negative serum and urine immunofixation with normalized FLC ratio (FLCR) as per current validated criteria^2^. The amyloid-forming light chain was determined by tissue typing (immuno-electron microscopy or mass spectrometry) in all subjects. Patients whose post-treatment FLCR was not within the reference range (0.26–1.65) but was inverted in favor of the non-amyloidogenic light chain, were considered to have a “normalized FLCR” for the purposes of aCR definition. The present population is also composed by those who did not fulfill the criteria for aCR but reached at least one of the following endpoints 6 months after treatment initiation: (1) iFLC <20 mg/L; (2) normalization of iFLC, (3) dFLC <10 mg/L, (4) normalization of FLCR. Moreover, we generated alternative definitions of CR based on the combination of negative serum and urine immunofixation with each of the three low-FLC endpoints, substituting normal-FLCR with iFLC <20 mg/L, normalization of iFLC, and dFLC <10 mg/L, respectively. In addition the remaining 156 patients who obtained at least a partial response to therapy at 6 months, but did not qualify for any of the proposed response categories were included, considered for additional analysis of survival and of the discriminating ability of alternative definitions of response.

Semi-automated serum and urine immunofixation was performed with commercial Hydragel 2IF/BJ(HR) kit on a Hydrasys apparatus (Sebia, Lisses, France). Serum FLC concentration was measured with a latex-enhanced immunoassay (The Binding Site, Birmingham, UK). Cardiac and renal responses were defined according to current criteria^2,10^ 6 months after treatment initiation, at the time of hematologic response assessment. All patients gave written informed consent for their clinical data to be used for research purposes. The Ethic committee of the Foundation IRCCS Policlinico San Matteo, Pavia (Italy) approved the study.

Fischer exact test or Mann–Whitney test were used to compare categorical and continuous variables, respectively. Overall survival (OS) and time to next line of therapy or death (TNTD) were calculated from the time of hematologic response assessment (6 months after treatment initiation) and compared by log-rank test. We tested the ability of alternative definitions of aCR to discriminate patients who survived more than 2 years by means of a Receiver Operating Characteristic (ROC) analysis based, calculating areas under ROC curves (AUC) and 95% confidence intervals (95% CI). Values range from 0.5 to 1.0, with 0.5 representing random chance and higher values indicating better discriminating ability.

## Results

Four-hundred thirty-four patients were included and distributed in five groups (Table 1): (a) patients in aCR (aCR group, *n* = 161), and subjects who did not qualify for aCR, namely (b) patients whose post-treatment iFLC was <20 mg/L (iFLC20 group, *n* = 66), (c) subjects who obtained normalization of iFLC (normal-iFLC group, *n* = 114), (d) patients whose post-treatment dFLC was <10 mg/L (dFLC10 group, *n* = 144), and (e) patients whose FLCR normalized (normal-FLCR group, *n* = 220). In the aCR group 26 patients had an FLCR that was not within the reference limit but favored the non-amyloidogenic light chain and were classified as aCR according to standard criteria. Patients who did not qualify for aCR could be included in all the groups they qualified for, resulting in a partial overlap of patients included in the non-aCR groups (Table 2). Importantly, only four patients who did not qualify for aCR, but were classified in groups b, c, or d, had negative serum and urine IFE. In these subjects the reason for not qualifying for aCR was an abnormal FLCR only. The clinical characteristics of these four patients are reported in Table 3.

**Table 1:** Patients characteristics.

	Complete response (*N* = 161) *N* (%)—median (IQR)	No complete response			
		iFLC < 20 mg/L (*N* = 66) *N* (%)—median (IQR)	Normal-iFLC (*N* = 114) *N* (%)—median (IQR)	dFLC < 10 mg/L (*N* = 144) *N* (%)—median (IQR)	Normal-FLCR (*N* = 220) *N* (%)—median (IQR)
Age, years	64 (57–69)	63 (56–70)	64 (55–70)	63 (56–68)	63 (55–68)
Male sex	87 (54)	29 (44)	58 (51)	83 (57)	133 (60)
Organ involvement					
Heart, kidney	110 (68)/115 (71)	46 (69)/44 (66)	79 (69)/76 (67)	107 (74)/103 (71)	165 (75)/158 (72)
Liver, soft tissue	15 (9)/24 (15)	6 (9)/5 (7)	9 (8)/14 (12)	20 (14)/19 (13)	28 (13)/29 (13)
PNS, ANS	17 (11)/11 (7)	3 (4)/4 (6)	12 (10)/5 (4)	8 (5)/7 (4)	18 (8)/20 (9)
Cardiac stage					
I/II	32 (20)/78 (49)	16 (24)/29 (47)	30 (26)/49 (43)	28 (20)/72 (49)	40 (19)/108 (48)
IIIa/IIIb	38 (24)/13 (7)	9 (13)/10 (15)	23 (21)/12 (10)	28 (20)/16 (11)	50 (23)/22 (10)
Renal stage					
I, II,	76 (47)/61 (38)	30 (45)/32 (49)	53 (46)/52 (45)	54 (38)/62 (43)	82 (37)/101 (47)
III, dialysis	24 (15)/0 (0)	2 (3)/2 (3)	7 (6)/2 (3)	20 (14)/8 (5)	34 (15)/3 (1)
eGFR < 30 mL/min	27 (17)	7 (10)	16 (14)	34 (23)	49 (22)
Intact-MC*	68 (42)	44 (66)	81 (71)	102 (71)	135 (61)
Light chain only MC	93 (58)	22 (34)	33 (29)	42 (29)	85 (39)
Kappa:lambda	25 (22):136 (78)	13 (19):53 (81)	13 (11):101 (79)	24 (16):120 (84)	21 (19):199 (81)
dFLC, mg/L**	98 (25–225)	100 (54–280)	103 (54–271)	103 (54–271)	144 (72–351)
BMPC, %	10 (6–15)	11 (5–19)	10 (6–15)	11 (7–17)	10 (6–15)
Main treatment type	MDex 48 (30)	MDex 10 (15)	MDex 26 (21)	MDex 28 (20)	MDex 58 (26)
	B-based 89 (55)	B-based 51 (77)	B-based 77 (67)	B-based 93 (64)	B-based 122 (55)
Cardiac response	50 (50)	13 (34)	29 (44)	30 (35)	39 (29)***
Renal response	54 (53)	23 (53)	45 (47)	44 (47)	63 (42)***
Patients receiving 2nd-line therapy****	47 (29)	31 (46)	61 (39)	74 (51)	121 (55)

Cardiac stage based on troponins level and NT-proBNP: thresholds for cTnI (or hs-cTnI) and NT-proBNP are <0.1 ng/mL (<77 ng/L), and <332 ng/L, respectively. Stage III cardiac involvement is defined at a cTnI >0.1 ng/mL or a hs-cTnI>77 ng/L, and NT-proBNP >332 ng/L (provided their NT-proBNP is <8500 ng/L). Stage II patients have one value of either troponin or NT-proBNP above the thresholds. Stage I patients have troponin and NT-proBNP below the thresholds. Renal stage based on proteinuria and eGFR levels: thresholds for proteinuria >5 g/24 h and eGFR <50 mL/min per 1.73 m^2^. Stage I, both proteinuria ≤5 g/24 h and eGFR ≥50 mL/min per 1.73 m^2^; stage II, either proteinuria >5 g/24 h or eGFR <50 mL/min per 1.73 m^2^; stage III, both proteinuria >5 g/24 h and eGFR <50 mL/min per 1.73 m^2^.

Second-line treatment types in the overall population: bortezomib-based 73 (37%); lenalidomide and dexamethasone 38 (19%); melphalan and dexamethasone 27 (14%); thalidomide based 26 (13%); autologous stem cell transplant 18 (9%); pomalidomide and dexamethasone 4 (2%); rituximab based 3 (1.5%); daratumumab based 3 (1.5%); bendamustine and prednisone 2 (1%); ixazomib and dexamethasone 2 (1%).

*ANS* autonomic nervous system, *BMPC* bone marrow plasma cells, *eGFR* estimated glomerular filtration rate (according to CKD-EPI), *dFLC* difference between involved and uninvolved free light chains, *IFE* immunofixation, *MC* monoclonal component, *MDex* melphalan and dexamethasone, *B-based* bortezomib-based regimens, *PNS* peripheral nervous system.

*CR group vs. all the others, *P* < 0.001.

**CR vs. FLCR, *P* < 0.001.

***Complete response group vs. normal-FLCR, *P* < 0.05.

****Complete response group vs. all the other categories, *P* < 0.001.

**Table 2:** Hematologic and organ response in the different study groups.

	Complete response (*N* = 161) *N* (%)	No complete response			
		iFLC < 20 mg/L (*N* = 66) *N* (%)	Normal-iFLC (*N* = 114) *N* (%)	dFLC < 10 mg/L (*N* = 144) *N* (%)	Normal-FLCR (*N* = 220) *N* (%)
CR (*N* = 161)	**161** **(100)**	0 (0)	0 (0)	0 (0)	0 (0)
iFLC < 20 mg/L (*N* = 66)	87 (54)	**66** **(100)**	64 (97)	57 (86)	46 (70)
Normal-iFLC (*N* = 114)	86 (53)	64 (56)	**114** (**100)**	86 (75)	81 (71)
dFLC < 10 mg/L (*N* = 144)	103 (64)	57 (39)	86 (60)	**144 (100)**	104 (72)
Normal-FLCR (*N* = 220)	**161** **(100)**	46 (70)	81 (71)	104 (72)	**220** **(100)**
IFE serum and urine negative	**161** **(100)**	4 (6)	4 (3)	3 (2)	0 (0)

Numbers in parenthesis represent the percentage of the patients of the referred row in all cases with the exception of the last row in which those numbers refer to the single column.

*CR* complete response, *iFLC* involved free light chains, *dFLC* difference between involved and uninvolved free light chains, *IFE* immunofixation.

Bold values represent row/column match.

**Table 3:** Clinical data of patients with negative serum and urine immunofixation at evaluation of response but not qualifying for aCR due to abnormal FLCR.

	Baseline						After 6 months of therapy				
	MC type	eGFR, mL/min	iFLC, mg/L	U-FLC, mg/L	dFLC, mg/L	FLCR	eGFR, mL/min	iFLC, mg/L	U-FLC, mg/L	dFLC, mg/L	FLCR
ID1	LC-λ	77	96	2.00	94	0.02	71	18	1.26	17	0.07
ID2	LC-λ	81	47	11.75	36	0.25	45	8	1.92	6	0.24
ID3	IgAλ	75	102	6.12	95	0.06	106	8	1.12	7	0.14
ID4	LC-κ	51	612	19.00	593	32	77	8	3.68	5	2.17

*MC* monoclonal component type, *LC* light chain, *eGFR* estimated glomerular filtration rate, *iFLC* involved free light chain, *dFLC* difference between involved and uninvolved free light chains, *FLCR* free light chain ratio, *U-FLC* uninvolved FLC.

No significant difference was observed between the aCR and other groups in the type of organ involvement, cardiac and renal staging at baseline. In particular, no difference in median estimated glomerular filtration rate was seen, as well as in the percentage of patients with eGFR <30 mL/min in the different groups. In the overall population, first-line treatment was bortezomib-based in 261 (60%) subjects, oral melphalan plus dexamethasone in 128 (29%), immunomodulatory based in 21 (5%), autologous stem cell transplant in 14 (3%), and therapy for IgM clones in the remaining 10 (2%) subjects. No differences in the main treatment types used were detected among the subgroups (Table 1) and all patients received only one line of therapy before the assessment of response. Cardiac and renal response rates were significantly lower in the normal-FLCR group compared to the aCR group, whereas organ response rates were similar in all the other groups (Tables 1 and 2). In the whole cohort, 197 (45%) of patients required a second-line therapy during the follow-up. This was significantly less frequent in subjects who reached aCR compared to all the other response categories (Tables 1 and 2).

**Fig. 1:**
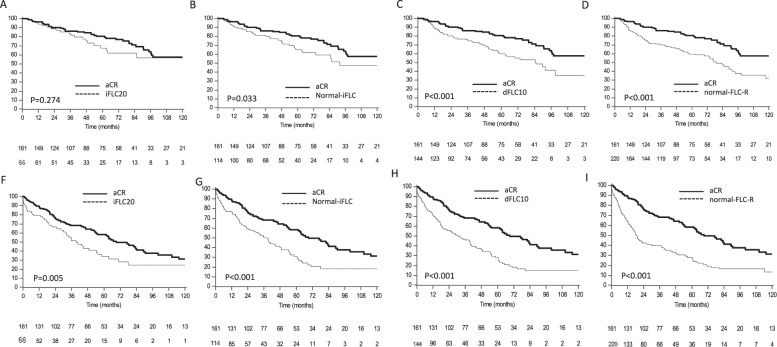
Overall survival and time to next treatment or death according to type of hematologic response in the whole cohort. **a** Overall survival in the aCR vs. iFLC20 group (*P* = 0.274) Bold line: patients in aCR group, median survival not reached (*N* = 161) Dotted line: patients in iFLC group, median survival not reached (*N* = 66). **b** Overall survival in the aCR vs. normal-iFLC groups (*P* = 0.033). Bold line: aCR group, median survival not reached (*N* = 161) Dotted line: normal-iFLC group, median survival 91 months (*N* = 114). **c** Overall survival in the aCR vs. dFLC10 groups (*P* < 0.001) Bold line: aCR group, median survival not reached (*N* = 161) Dotted line: dFLC10 group, median survival 85 months (*N* = 144). **d** Overall survival in the aCR vs. normal-FLCR groups (*P* < 0.001) Bold line: patients in aCR cohort, median survival not reached (*N* = 161) Dotted line: patients in normal-FLCR cohort, median survival 79 months (*N* = 220). **e** Time to next treatment or death in the aCR vs. iFLC20 groups (*P* = 0.005). Bold line: aCR group, median 69 months (*N* = 161) Dotted line: iFLC20 group, median 39 months (*N* = 66). **f** Time to next treatment or death in the aCR vs. normal-iFLC groups (*P* < 0.001) Bold line: aCR group, median 69 months (*N* = 161) Dotted line: normal-iFLC group, median 39 months (*N* = 114). **g** Time to next treatment or death in the aCR vs. dFLC10 groups (P < 0.001). Bold line: aCR group, median 69 months (*N* = 161) Dotted line: dFLC10 group, median 32 months (*N* = 144). **h** Time to next treatment or death in the aCR cohort vs. normal-FLCR groups (*P* < 0.001) Bold line: aCR group, median 69 months (*N* = 161) Dotted line: normal-FLCR group, median 18 months (*N* = 220).

The median follow-up of living patients was 55 months and median OS in the entire cohort was 93 months. Patients who achieved aCR had a significantly longer OS compared to patients in the normal-iFLC, dFLC10, and normal-FLCR groups, whereas, survival of subjects in the iFLC20 cohort was similar to that of aCR patients (Fig. 1a–d). Median OS was not reached in the aCR (80% alive at 5 years) and in the iFLC20 (70% alive at 5 years, *P* = 0.273 compared to aCR) groups, while it was 91 months in the normal-iFLC cohort (*P* = 0.033 compared to aCR), 85 months in the dFLC10 group (*P* < 0.001 compared to aCR), and 79 months in the normal-FLCR cohort (*P* < 0.001 compared to aCR). Median TNTD (Fig. 1e–h) in the whole cohort was 31 months, and was significantly longer in patients who obtained aCR (median 69 months) compared to the iFLC20 cohort (median 39 months, *P* = 0.005), the normal-iFLC group (median 39 months, *P* < 0.001), the dFLC10 group (median 32 months, *P* < 0.001), and the normal-FLCR group (median 18 months, *P* < 0.001).

We then analyzed the outcome of all the proposed response categories in the all cohort of patients who obtained very good partial response (VGPR) and partial response (PR) at 6 months, but did not qualify for aCR or any of the low-FLC response categories. The OS of patients who attained any of the low-FLC endpoints but not aCR was not significantly longer compared to those of subjects in VGPR, with the exception of patients in the iFLC20 group, whose OS was longer than that of subjects attaining VGPR (Supplemental Fig. 1).

**Fig. 2:**
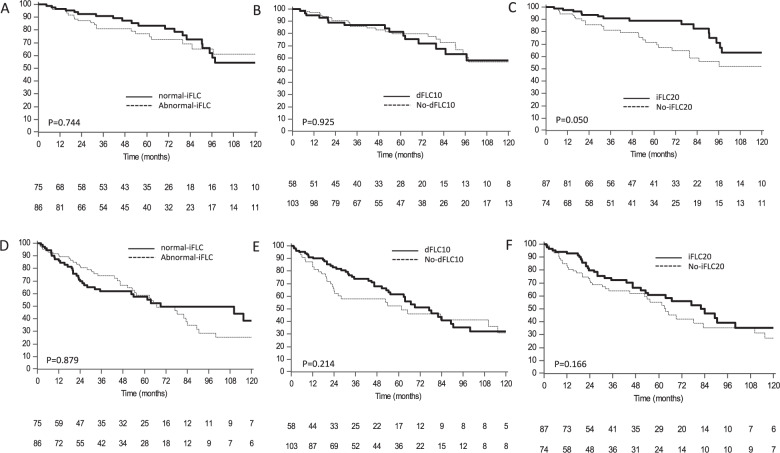
Overall survival and time to next treatment or death according to type of hematologic response in patients who reached aCR. **a** Overall survival according to iFLC normalization in patients in aCR (*P* = 0.744). Bold line: patients in aCR with normalized-iFLC, median survival 133 months (*N* = 86). Dotted line: patients in aCR without normalized-iFLC, median survival not reached (*N* = 75). **b** Overall survival according to dFLC < 10 mg/L in patients in aCR (*P* = 0.925). Bold line: patients in aCR with dFLC <10 mg/L, median survival not reached (*N* = 103). Dotted line: patients in aCR without dFLC <10 mg/L, median survival not reached (*N* = 58). **c** Overall survival according to iFLC < 20 mg/L in patients in aCR (*P* = 0.050). Bold line: patients in aCR with iFLC<20 mg/L, median survival not reached (*N* = 87). Dotted line: patients in aCR without iFLC<20 mg/L, median survival not reached (*N* = 74). **d** Time to next treatment or death according to iFLC normalization in patients in aCR (*P* = 0.879). Bold line: patients in aCR with normalized-iFLC, median 69 months (*N* = 86). Dotted line: patients in aCR without normalized-iFLC, median 76 months (*N* = 75). **e** Time to next treatment or death according to dFLC < 10 mg/L in patients in aCR (*P* = 0.214). Bold line: patients in aCR with dFLC <10 mg/L, median 76 months (*N* = 103). Dotted line: patients in aCR without dFLC <10 mg/L, median 61 months (*N* = 58). **f** Time to next treatment or death according to iFLC20 in patients in aCR (*P* = 0.166). Bold line: patients in aCR with iFLC<20 mg/L, median 82 months (*N* = 87). Dotted line: patients in aCR without iFLC<20 mg/L, median 63 months (*N* = 75).

We then analyzed whether, among subjects who qualified for aCR, achieving a profound FLC reduction could discriminate subgroups with longer survival. In the aCR group, 87 subjects (54%) also attained iFLC <20 mg/L, 86 subjects (53%) also reached iFLC normalization, and 103 patients (64%) also achieved dFLC <10 mg/L (Table 2). There was no difference in OS (Fig. 2a, b) and TNTD (Fig. 2d, e) between subgroups of patients in aCR according to normal-iFLC or dFLC10 response. However, patients in aCR who also attained iFLC <20 mg/L had a modest, not significant, OS (median not reached in both groups, *P* = 0.050, Fig. 2c) and TNTD advantage (median 82 vs. 63 months *P* = 0.166, Fig. 2f). Median OS and TNTD in aCR patients with normal vs. abnormal iFLC were not reached vs. 133 months (*P* = 0.744) and 69 vs. 76 months (*P* = 0.879), respectively. Patients in aCR with dFLC <10 mg/L had similar OS (not reached in both groups, *P* = 0.925) and TNTD (76 vs. 61 months, *P* = 0.214) compared to the remaining subjects in aCR.

In the aCR group, no significant difference in cardiac (47% vs. 49%, *P* = 0.428) and renal (54% vs. 51%, *P* = 0.381) response rates was observed in patients who obtained iFLC <20 mg/L compared to those who did not. Cardiac (51% vs. 47%, *P* = 0.346) and renal (57% vs. 48%, *P* = 0.178) response rates were similar in aCR patients who obtained iFLC normalization compared to those who did not. Finally, in patients who attained aCR, reaching a dFLC concentration <10 mg/L was not associated with significantly different cardiac (51% vs. 47%, *P* = 0.340) or renal (52% vs. 53%, *P* = 0.444) response rates.

An analysis including only bortezomib-treated patients (*N* = 261), showed significantly longer TNTD for patients in aCR compared to all other groups, and better OS compared to the dFLC10 and normal-FLCR cohorts, while the OS survival advantage over the iFLC20 and normal-iFLC groups did not reach statistical significance (Supplemental Fig. 2). In bortezomib-treated patients who attained aCR, no significant advantage in OS and TNTD was seen in patients reaching any FLC endpoint (Supplemental Fig. 3).

An analysis including only patients with a baseline eGFR >30 mL/min (*N* = 357), showed significantly longer TNTD for patients in aCR compared to all other groups, and better OS compared to the all the other cohorts with the exception of the iFLC20 group (Supplemental Fig. 4). In patients with a baseline eGFR >30 mL/min who attained aCR, no significant advantage in OS and TNTD was seen in patients reaching any FLC endpoints (not shown).

Finally, we assed whether alternative definitions of CR, all based on negative serum and urine IF and obtained substituting normal-FLCR with each of the low-FLC endpoints could better discriminate patients who survived longer than 2 years in the population including all the patients who attained at least partial response at 6 months after treatment initiation. None of the alternative CR definitions had a better performance than standard aCR, which had the higher AUC value (0.705, 95% CI 0.662–0.745), which was 0.702 (95% CI 0.656–0.746) for combination of negative serum and urine IFE with iFLC <20 mg/L, 0.696 (95%CI 0.651–0.739) for combination of negative serum and urine IFE with dFLC <10 mg/L, and 0.697 (95% CI 0.651–0.741) for combination of negative serum and urine IFE with normal-iFLC.

## Discussion

The present study is based on the largest patient population (1378 consecutive subjects) systematically searched for individuals with profound hematologic response (*N* = 434 patients). We observed that patients attaining profound FLC responses but who do not qualify for aCR have shorter survival compared to subjects who reach aCR. We also found that amongst patients in aCR, those attaining an iFLC level <20 mg/L may have a modest OS advantage. The very similar results obtained by the Boston University group in a population of patients undergoing autologous stem cell transplantation corroborate our findings and provide an external validation. In addition, we remarkably observed that patients who attained a profound reduction of the FLC levels after treatment but not aCR had a positive serum and/or immunofixation (performed with a standard, commercial, semi-automated method) in >90% of cases. This strongly indicates that abnormal FLCR only exceptionally is the reason why patients who attain low-FLC levels do not qualify for aCR. The great majority of them still have detectable monoclonal component in serum and/or urine. This emphasizes that FLC quantification cannot rule out the persistence of small, non-measurable amounts of monoclonal protein that can still affect outcomes.

In our population, patients who achieved aCR had significantly longer OS and TNTD than subjects who attained iFLC normalization, a dFLC level <10 mg/L, or a normalized FLCR, and significantly longer TNTD compared to patients who reached an iFLC concentration <20 mg/L. Amongst patients who attained aCR, those who also reached an iFLC concentration <20 mg/L tended to have longer OS and TNTD. In addition, obtaining a reduction of iFLC below 20 mg/L but not aCR defined a subgroup of subjects with a significantly longer overall survival compared to VGPR. Taken together these observations suggest that the definition of aCR could become even more stringent but should always include negative serum and urine IFE. Importantly, in the subgroup analysis of patients homogenously treated with bortezomib-based regimens, patients who reached aCR had longer OS and TNTD compared to subjects attaining low-FLC endpoints but not aCR. In addition, the exclusion of patients with a moderate and severe renal dysfunction (eGFR < 30 mL/min), known to affect the FLC levels and ratio, did not influence the main results of our analysis. Finally, we were unable to prove that alternative definition of CR, substituting FLCR with each of the low-FLC endpoints, could improve the ability of the current definition of aCR to discriminate patients who survive longer.

Further investigations in patients treated with novel agents and evaluated with modern tools to detect clonal disease are warranted. For instance, the use of immunotherapies, such as daratumumab, may affect the concentration of uninvolved FLC, interfering with dFLC and FLCR calculation. In addition, patients whose amyloidogenic monoclonal component is a complete immunoglobulin of the same isotype of the therapeutic monoclonal antibody require specific consideration. In these subjects, specific methods for “antibody cleaning” during immunofixation can be developed^11,12^. Alternatively, mass-spectrometry approaches^13^ can unequivocally distinguish the disease-specific immunoglobulin from therapeutic monoclonal antibodies. However, the possibility of substituting IFE with mass spectrometry in the definition of CR in AL amyloidosis still needs large validation studies. For now, aCR should be the goal of therapy if tolerability and patient frailty allow. Still, ~30% of patients in aCR relapse and eventually die. New sensitive tools to detect residual clonal disease (e.g., mass-spectrometry on serum and urine, next-generation sequencing and flow cytometry on bone marrow)^14–16^ are needed to identify these patients and intervene before relapse. As previously reported by the Mayo investigators, the persistence of monotypic plasma cells in the bone marrow at the end of therapy, correlates with a shorter overall survival, progression-free survival and could hinders organ recovery in patients in complete response^17^. Our group and the Boston University investigators reported a possible correlation between MRD negativity and higher probability of organ response after treatment in AL amyloidosis^18,19^.

In conclusion, available data do not support an update of response criteria based on FLC measurement and aCR should remain the goal of therapy if tolerability allows. Negative serum and urine immunofixation should remain part of the definition of CR. Large international collaborative studies are needed in order to improve current response criteria and validate novel definitions based on new technologies.

## Supplementary information

Supplementary materials

## References

[1] Merlini G (2018). Systemic immunoglobulin light chain amyloidosis. Nat. Rev. Dis. Primers.

[2] Palladini G (2012). New criteria for response to treatment in immunoglobulin light chain amyloidosis based on free light chain measurement and cardiac biomarkers: impact on survival outcomes. J. Clin. Oncol..

[3] Milani P (2017). Patients with light-chain amyloidosis and low free light-chain burden have distinct clinical features and outcome. Blood.

[4] Dittrich T (2017). AL amyloidosis patients with low amyloidogenic free light chain levels at first diagnosis have an excellent prognosis. Blood.

[5] Sidana S (2018). Clinical presentation and outcomes in light chain amyloidosis patients with non-evaluable serum free light chains. Leukemia.

[6] Manwani R (2019). A prospective observational study of 915 patients with systemic AL amyloidosis treated with upfront bortezomib. Blood.

[7] Muchtar E (2019). Optimizing deep response assessment for AL amyloidosis using involved free light chain level at end of therapy: failure of the serum free light chain ratio. Leukemia.

[8] Sidana S (2019). Revisiting complete response in light chain amyloidosis. Leukemia.

[9] Godara AR (2019). In systemic light-chain amyloidosis complete and very good partial responses are not enough: involved free light chain (iFLC) levels <10mg/L are associated with optimal long-term survival. Blood.

[10] Palladini G (2014). A staging system for renal outcome and early markers of renal response to chemotherapy in AL amyloidosis. Blood.

[11] McCudden C (2016). Monitoring multiple myeloma patients treated with daratumumab: teasing out monoclonal antibody interference. Clin. Chem. Lab. Med..

[12] Thoren KL, Pianko MJ, Maakaroun Y, Landgren CO, Ramanathan LV (2019). Distinguishing drug from disease by use of the hydrashift 2/4 daratumumab assay. J. Appl. Lab. Med..

[13] Moore LM, Cho S, Thoren KL (2019). MALDI-TOF mass spectrometry distinguishes daratumumab from M-proteins. Clin. Chim. Acta.

[14] Milani P (2017). The utility of MASS-FIX to detect and monitor monoclonal proteins in the clinic. Am. J. Hematol..

[15] Milani P, Merlini G, Palladini G (2018). What does minimal residual disease mean in AL amyloidosis? Expert Opinion on Orphan. Drugs.

[16] Dispenzieri A (2020). Blood mass spectrometry detects residual disease better than standard techniques in light-chain amyloidosis. Blood Cancer J..

[17] Muchtar E (2017). The prognostic value of multiparametric flow cytometry in AL amyloidosis at diagnosis and at the end of first-line treatment. Blood.

[18] Palladini G (2016). Persistence of minimal residual disease by multiparameter flow cytometry can hinder recovery of organ damage in patients with AL amyloidosis otherwise in complete response. Blood.

[19] Staron A (2020). Assessment of minimal residual disease using multiparametric flow cytometry in patients with AL amyloidosis. Blood Adv..

