# Supplementary material for: Indicators of profound hematologic response in AL amyloidosis: complete response remains the goal of therapy
Source: Blood Cancer J. 2020 Sep 1;10(8):90. doi: 10.1038/s41408-020-00355-6 (PMC7463008; doi:10.1038/s41408-020-00355-6)

Supplementary informations:

Indicators of profound hematologic response in AL amyloidosis: complete response remains the goal of therapy

Paolo Milani, Marco Basset, Mario Nuvolone, Francesca Benigna, Lara Rodigari, Francesca Lavatelli, Andrea Foli, Giampaolo Merlini, Giovanni Palladini

Amyloidosis Research and Treatment Center, Foundation IRCCS Policlinico San Matteo, and Department of Molecular Medicine, University of Pavia, Pavia, Italy.

Corresponding author:
Giovanni Palladini, M.D., Ph.D.
Amyloidosis Research and Treatment Center, Fondazione IRCCS Policlinico San Matteo
Viale Golgi, 19 – 27100 Pavia, Italy
e-mail: giovanni.palladini@unipv.it

Supplementary Figure 1.

Panel A. Overall survival according CR vs. iFLC<20 mg/L vs. VGPR vs. PR

Bold line: patients in aCR, median survival not reached (N=161)

Dashed line: patients in iFLC<20 mg/L, median survival not reached (N=66)

Dotted line: patients in VGPR, median survival 83 months (N=213)

Other line: patients in PR, median survival 48 months (N=150)

Panel B. Overall survival according CR vs. iFLCnormal vs. VGPR vs. PR

Bold line: patients in aCR, median survival not reached (N=161)

Dashed line: patients in iFLCnormal, median survival 91 months (N=114)

Dotted line: patients in VGPR, median survival 83 months (N=165)

Other line: patients in PR, median survival 48 months (N=150)

Panel C. Overall survival according CR vs. dFLC<10 mg/L vs. VGPR vs. PR

Bold line: patients in aCR, median survival not reached (N=161)

Dashed line: patients in dFLC<10 mg/L, median survival 85 months (N=144)

Dotted line: patients in VGPR, median survival 86 months (N=135)

Other line: patients in PR, median survival 48 months (N=150)

Panel D. Overall survival according CR vs. normal-FLCR vs. VGPR vs. PR

Bold line: patients in aCR, median survival not reached (N=161)

Dashed line: patients in normal-FLCR, median survival 79 months (N=220)

Dotted line: patients in VGPR, median survival 86 months (N=81)

Other line: patients in PR, median survival 50 months (N=128)

Supplementary Figure 1.

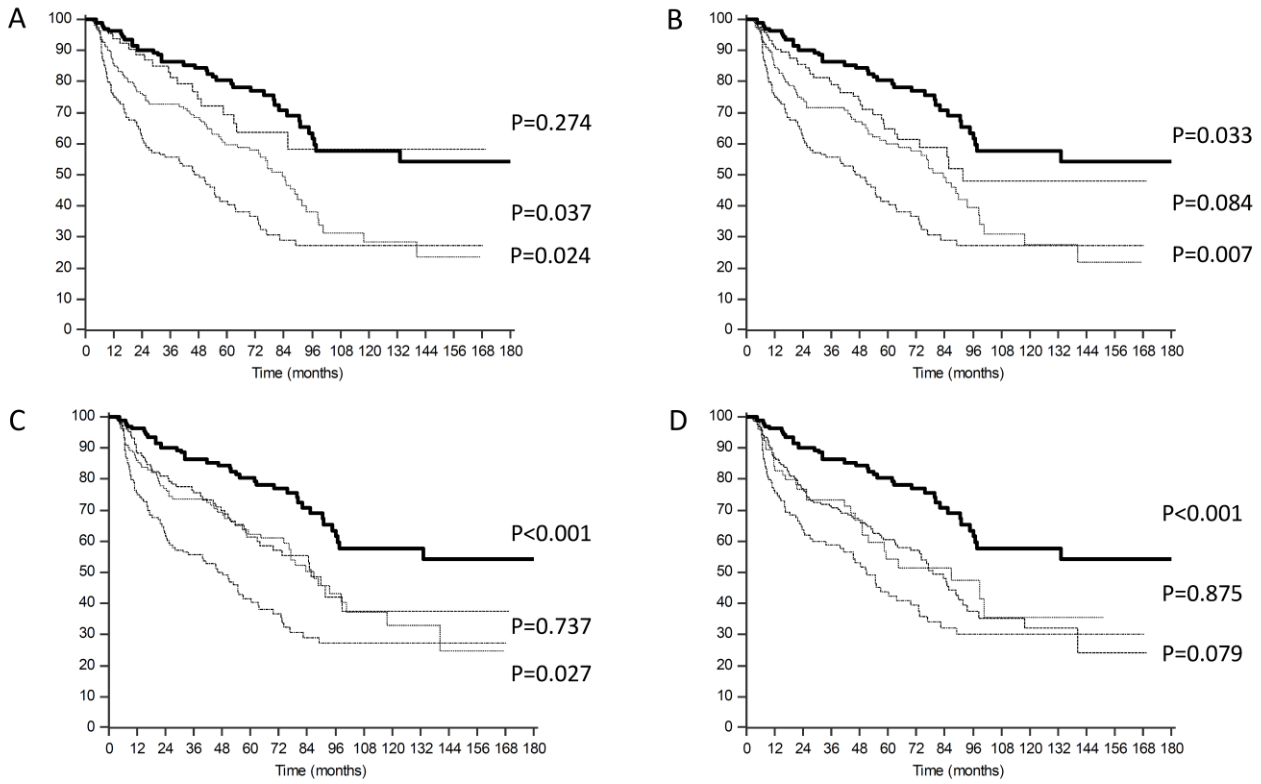

Supplementary Figure 2. Overall survival and time to next treatment or death according to type of hematologic response in patients treated with bortezomib based regimen.

Panel A. Overall survival in the aCR vs. iFLC20 group (P=0.228)

Bold line: patients in aCR group, median survival not reached (N=94)

Dotted line: patients in iFLC20 group, median survival not reached (N=52)

Panel B. Overall survival in the aCR vs. normal-iFLC groups (P=0.081)

Bold line: aCR group, median survival not reached (N=94)

Dotted line: normal-iFLC group, median survival not reached (N=81)

Panel C. Overall survival in the aCR vs. dFLC10 groups (P=0.010)

Bold line: aCR group, median survival not reached (N=94)

Dotted line: dFLC10 group, median survival not reached (N=100)

Panel D. Overall survival in the aCR vs. normal-FLCR groups (P=0.004)

Bold line: patients in aCR cohort, median survival not reached (N=94)

Dotted line: patients in normal-FLCR cohort, median survival not reached (N=134)

Panel E. Time to next treatment or death in the aCR vs. iFLC20 groups (P=0.017)

Bold line: aCR group, median 83 months (N=94)

Dotted line: iFLC20 group, median 39 months (N=52)

Panel F. Time to next treatment or death in the aCR vs. normal-iFLC groups (P<0.001)

Bold line: aCR group, median 83 months (N=94)

Dotted line: normal-iFLC group, median 33 months (N=81)

Panel G. Time to next treatment or death in the aCR vs. dFLC10 groups (P<0.001)

Bold line: aCR group, median 83 months (N=94)

Dotted line: dFLC10 group, median 30 months (N=100)

Panel H. Time to next treatment or death in the aCR cohort vs. normal-FLCR groups (P<0.001)

Bold line: aCR group, median 83 months (N=94)

Dotted line: normal-FLCR group, median 19 months (N=134).

Supplementary Figure 2.

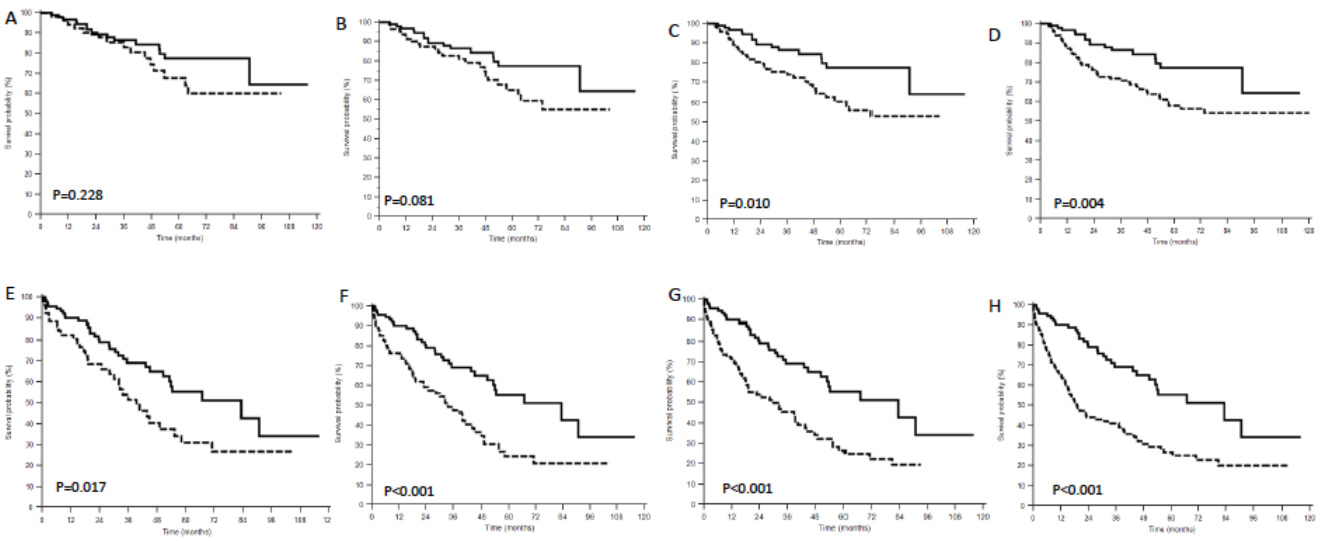

Supplementary Figure 3. Overall survival and time to next treatment or death according to type of hematologic response in patients in aCR treated with a bortezomib based regimen.

Panel A. Overall survival according to iFLC<20 mg/L in patients in aCR (P=0.089)

Bold line: patients in aCR with iFLC<20 mg/L, median survival not reached (N=60)

Dotted line: patients in aCR without iFLC<20 mg/L, median survival not reached (N=34)

Panel B. Overall survival according to iFLC normalization in patients in aCR (P=0.941)

Bold line: patients in aCR with normalized iFLC, median survival not reached (N=55)

Dotted line: patients in aCR without normalized iFLC, median survival 133 months (N=39)

Panel C. Overall survival according to dFLC <10 mg/L in patients in aCR (P=0.851)

Bold line: patients in aCR with dFLC <10 mg/L, median survival not reached (N=65)

Dotted line: patients in aCR without dFLC <10 mg/L, median survival not reached (N=29)

Panel D. Time to next treatment or death according to iFLC20 in patients in aCR (P=0.526)

Bold line: patients in aCR with iFLC<20 mg/L, median 90 months (N=60)

Dotted line: patients in aCR without iFLC<20 mg/L, median 83 months (N=34)

Panel E. Time to next treatment or death according to iFLC normalization in patients in aCR (P=0.556)

Bold line: patients in aCR with normalized iFLC, median 66 months (N=55)

Dotted line: patients in aCR without normalized iFLC, median not reached (N=39)

Panel F. Time to next treatment or death according to dFLC <10 mg/L in patients in aCR (P=0.246)

Bold line: patients in aCR with dFLC <10 mg/L, median 90 months (N=65)

Dotted line: patients in aCR without dFLC <10 mg/L, median 83 months (N=29)

Supplementary Figure 3

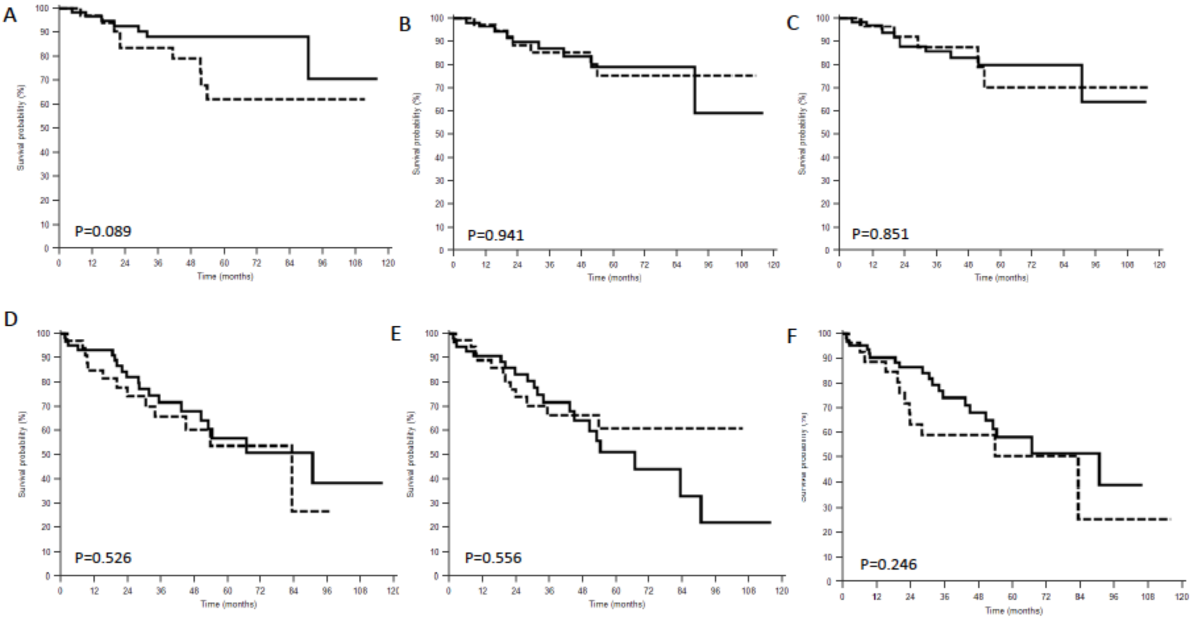

Supplementary Figure 4. Overall survival and time to next treatment or death according to type of hematologic response in patients with an eGFR >30 mg/L.

Panel A. Overall survival in the aCR vs. iFLC20 group (P=0.379)

Bold line: patients in aCR group, median survival not reached (N=134)

Dotted line: patients in iFLC group, median survival not reached (N=60)

Panel B. Overall survival in the aCR vs. normal-iFLC groups (P=0.048)

Bold line: aCR group, median survival not reached (N=134)

Dotted line: normal-iFLC group, median survival not reached (N=102)

Panel C. Overall survival in the aCR vs. dFLC10 groups (P=0.035)

Bold line: aCR group, median survival not reached (N=134)

Dotted line: dFLC10 group, median survival 89 months (N=113)

Panel D. Overall survival in the aCR vs. normal-FLCR groups (P<0.001)

Bold line: patients in aCR cohort, median survival not reached (N=134)

Dotted line: patients in normal-FLCR cohort, median survival 89 months (N=180)

Panel E. Time to next treatment or death in the aCR vs. iFLC20 groups (P=0.007)

Bold line: aCR group, median 78 months (N=134)

Dotted line: iFLC20 group, median 40 months (N=60)

Panel F. Time to next treatment or death in the aCR vs. normal-iFLC groups (P<0.001)

Bold line: aCR group, median 78 months (N=134)

Dotted line: normal-iFLC group, median 39 months (N=102)

Panel G. Time to next treatment or death in the aCR vs. dFLC10 groups (P<0.001)

Bold line: aCR group, median 78 months (N=134)

Dotted line: dFLC10 group, median 32 months (N=113)

Panel H. Time to next treatment or death in the aCR cohort vs. normal-FLCR groups (P<0.001)

Bold line: aCR group, median 78 months (N=134)

Dotted line: normal-FLCR group, median 19 months (N=180)

Supplementary figure 4

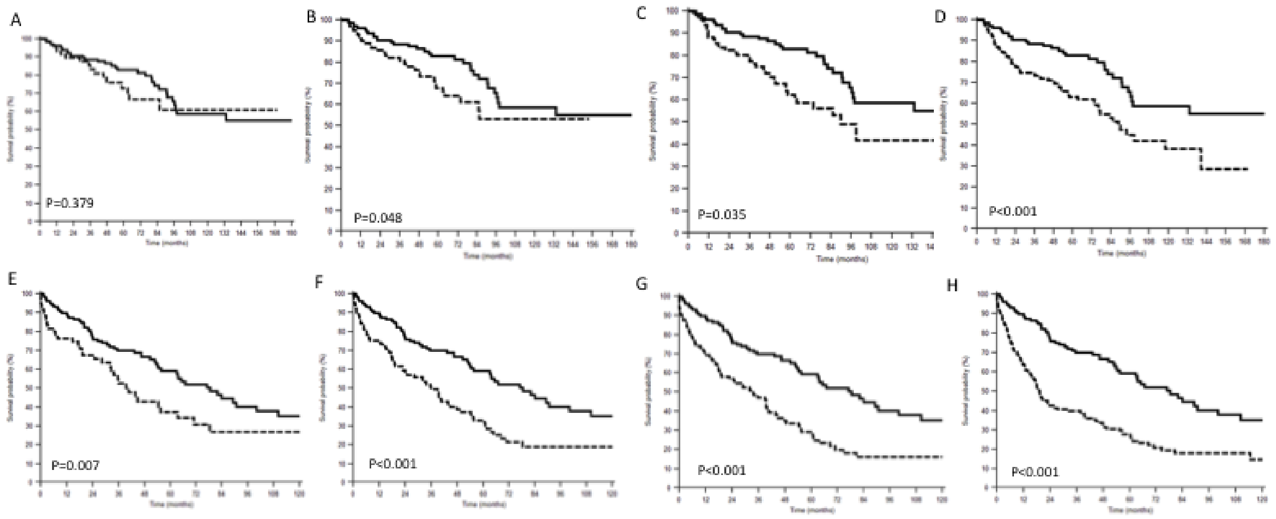

Supplement: Supplementary file 1 — Supplementary materials [file 41408_2020_355_MOESM1_ESM.pdf]
